# Minimal Change Disease in an Adult With Secondary Hypogammaglobulinemia: A Case Report

**DOI:** 10.7759/cureus.80555

**Published:** 2025-03-14

**Authors:** Gautam Agrawal, Bhawna Agarwal

**Affiliations:** 1 Nephrology, Independence Health System, Greensburg, USA; 2 Internal Medicine, University of Pittsburgh Medical Center McKeesport Hospital, McKeesport, USA

**Keywords:** hypogammaglobulinemia, minimal change nephrotic syndrome, recurrent pulmonary infection, steroid-dependent minimal change disease, steroid-sensitive nephrotic syndrome

## Abstract

Minimal change disease (MCD) is a common cause of nephrotic syndrome, particularly in children, but it also affects adults, albeit in smaller proportions. MCD presents with edema, severe proteinuria, hypoalbuminemia, and hyperlipidemia. MCD can lead to secondary hypogammaglobulinemia and an increased risk of infections by causing increased loss of immunoglobulins in the urine, impaired IgG synthesis, and from the immunosuppressive therapies used to treat MCD. The mainstay treatment for MCD is corticosteroids, which induce remission in most cases. However, some patients require other immunosuppressive therapies due to persistent relapses or resistance to standard treatment. This case report presents a 60-year-old woman with recurrent infections who was found to have hypogammaglobulinemia, with further workup showing nephrotic syndrome due to MCD. Her immunoglobulin levels normalized as her proteinuria levels improved with the treatment of her MCD.

## Introduction

Minimal change disease (MCD) is the most common cause of nephrotic syndrome in children but also accounts for a small proportion of cases in adults (10-15%) [1,2]. The exact cause of MCD remains unclear; however, evidence indicates that systemic T cell dysfunction leads to the production of a glomerular permeability factor. This factor impacts the glomerular capillary wall, causing proteinuria and effacement of the foot processes. MCD can be primary (idiopathic), with no clear inciting factors, or it can be associated with secondary causes, including drugs, neoplasms, infections, and allergies. MCD usually presents with the sudden onset of nephrotic syndrome symptoms, including edema, weight gain, proteinuria (albuminuria of more than 3.5 grams in a 24-hour period), hypoalbuminemia, and hyperlipidemia. MCD can also be associated with secondary hypogammaglobulinemia (SHG) [3], as well as a higher risk of infections and thromboembolism. Unlike in children, a kidney biopsy is needed to establish the diagnosis of MCD in adults [4]. On kidney biopsy, the glomeruli appear normal by light microscopy, with no evidence of immune complex deposition, and electron microscopy shows diffuse effacement of the podocyte foot processes. The mainstay of treatment is corticosteroids, which induce remission in most cases. However, steroid-sparing immunosuppressive therapies, such as cyclophosphamide, calcineurin inhibitors, mycophenolate mofetil, or rituximab, may be required in patients who experience frequent relapses or become steroid-dependent [4].

Secondary hypogammaglobulinemia requires detailed evaluation to rule out primary immunodeficiencies and to establish its potential cause, as it can be associated with nephrotic syndrome or medications used for treatment, necessitating a multidisciplinary approach [3]. This case report presents the clinical course and management of an adult patient with MCD and SHG, and demonstrates the normalization of IgG with effective treatment of MCD.

## Case presentation

A 60-year-old woman who had pertinent past medical history of hypertension, discoid lupus (based on skin biopsy), chronic nonsteroidal anti-inflammatory drugs (NSAIDs) use (used ibuprofen but stopped in 2018), atrial fibrillation, asthma, chronic obstructive pulmonary disease (COPD), and hypothyroidism. Her father had chronic kidney disease with unclear etiology. There was no known family history of autoimmune kidney disease. She started having worsening edema along with a 40-pound weight gain over three months since October 2022. She was started on loop diuretics with minimal improvement. Her urinalysis showed proteinuria, for which she was referred to nephrology in early 2023. 

On the initial exam, her weight was 191 lbs. Her BP was 126/68 mm hg. She was hypervolemic with 2+ lower extremity edema along with flank edema. Her lungs were clear to auscultation bilaterally. Her skin showed no malar rash or petechiae. As shown in Table 1, her lab results were significant for nephrotic syndrome, with a 24-hour urine protein of 13.5 g and albumin of 1.8. Her renal function was normal, with a creatinine of 0.7 mg/dl. Her serologies showed normal complements, negative antinuclear antibody (ANA), and negative double-stranded DNA. Her serum protein electrophoresis was negative for monoclonal protein. Her hepatitis B surface antigen and hepatitis C antibody were non-reactive. Her hemoglobin A1c was 6.1%.

**Table 1:** Laboratory values. LDL: low-density lipoprotein, TG: triglycerides, BUN: blood urea nitrogen, ANA: antinuclear antibody.

Lab tests	Results	Reference range	Units
WBC	6.71	3-10.2	Thousands/µL
Hemoglobin	12.1	11.7-15.8	gm/dl
Platelets	391	130-400	Thousands/µL
24 Hour protein	13,500	<150	mgs
Albumin	1.8	3.5-5.7	gm/dl
BUN	16	7-25	mg/dl
Creatinine	0.6	0.6-1.2	mg/dl
ANA	0.1	<0.8-Negative	N/A
Anti dsDNA	6.99	<25-Negative	IU/ml
HbA1c	6.1	5.7-6.4 (Prediabetic)	%
Hep Bs AG	Non-reactive	Non-reactive	N/A
Hep C Ab	Non-reactive	Non-reactive	N/A
LDL	120	<101	mg/dl
TG	218	<150	mg/dl

She had recurrent infections. Her notable infections were recurrent pneumonia, COVID-19, impetigo, shingles sinus infections, and recurrent episodes of COPD exacerbation in the last 15 months. She was diagnosed with hypogammaglobulinemia based on low IgG levels, as shown below in Table 2. Her IgM and IgA levels were within normal limits. Given her recurrent infections and hypogammaglobulinemia, she was referred to hematology. Her initial IgG level was 415 in February 2023, for which hematology initially recommended intravenous immunoglobulin (IVIG), but this could not be done due to financial issues.

**Table 2:** Laboratory values-immunoglobulin levels.

Lab tests	Results	Reference range	Units
IgG	415	635-1741	mg/dl
IgA	266	66-433	mg/dl
IgM	113	45-281	mg/dl

Meanwhile, she underwent a kidney biopsy that showed a relatively unremarkable appearance of the glomeruli by routine light microscopy and negative immunofluorescence studies but showed widespread podocyte foot process fusion (90%) on ultrastructural examination, which was consistent with minimal-change disease. She was started on 60 mg of prednisone, responded well, and went quickly into remission (urinalysis negative for proteinuria), following which steroids were tapered and stopped after 12 weeks. Unfortunately, she relapsed within two weeks of stopping steroids as she started having edema with urinalysis showing >1000 mg/dl of protein, and was restarted back on steroids by the end of July with slow taper over six months. She underwent remission (no proteinuria on urinalysis) within a week of starting steroids. 

**Figure 1:**
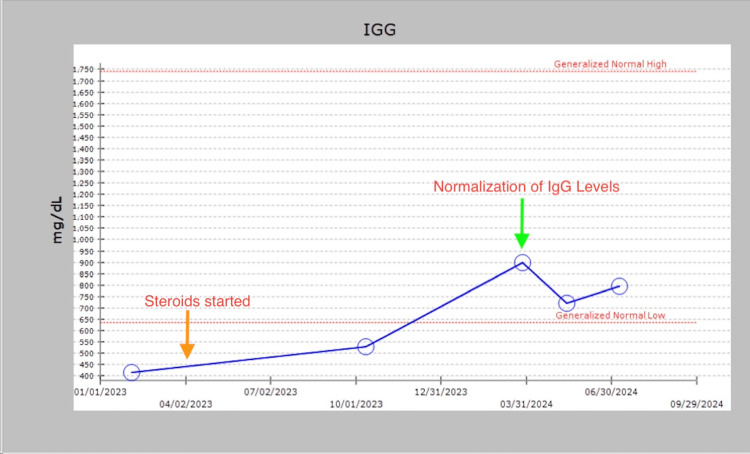
IgG level trends. Y-axis: IgG level (mg/dl) and X-axis: time (month/year). The orange arrow indicating when steroids started and the green arrow indicating the normalization of IgG levels.

Meanwhile, her IgG levels started improving slowly, and she had spontaneous remission by early 2024. Her IgG levels have remained within normal limits without needing any IVIG. Her levels indicated an initial 415 mg/dl in February 2023, as depicted in Figure 1. She was started on steroids in April 2023, as shown by the orange arrow in Figure 1, following which she went into remission. Her next IgG level improved to 530 mg/dl in October 2023 and then became normal to 899 mg/dl in March 2024 and has remained within normal limits.

Steroids were stopped, but she again relapsed after stopping them. She was deemed to be steroid dependent, and immunosuppressive medications were discussed and recommended with the patient, but she refused to start giving her prior recurrent infections. Fortunately, she remained in remission for the past six months at a low dose of prednisone 5 mg daily, and those are being further tapered with a plan to stop it. Since she has not had any infections in the past few months, along with improved immunoglobulin, she has agreed to start tacrolimus if she relapses again.

## Discussion

We present a case of a patient who presented with volume overload. The primary differentials in her case include heart failure, liver disease, kidney disease, or medication-related causes. Her echocardiogram showed a normal left ventricular ejection fraction with normal diastolic dysfunction. Her liver function tests were unremarkable. Her urinalysis showed proteinuria along with severe hypoalbuminemia, which suggested nephrotic syndrome. She was diagnosed with MCD and found to have SHG. 

This case highlights the challenges in managing MCD associated with SHG in an adult patient, particularly in those with recurrent relapses and steroid dependence. While most patients respond well to corticosteroid treatment, a considerable proportion experience relapses [1,2]. The risk of relapse is higher in patients with a higher baseline estimated glomerular filtration rate (eGFR), and early relapses are more common in females [5]. Long-term steroid use can lead to complications such as osteoporosis, diabetes, hypertension, and cataracts and is associated with considerable morbidity, necessitating careful monitoring and consideration of steroid-sparing agents [2]. Most data for the management of MCD are derived from clinical trials in children and observational studies performed in patients of various ages. There are only a few studies in adults with MCD [6]. Data from some studies suggest using alternative immunosuppressive therapies in patients at high risk of complications from steroids or for steroid-dependent MCD [7].

MCD can be associated with SHG due to protein loss in urine, impaired IgG synthesis, and immunosuppressive therapies used to treat MCD. A detailed evaluation and multidisciplinary approach are required for SHG associated with MCD. Serum IgG levels normalize with the remission of MCD [8]. There is insufficient evidence to recommend the routine use of IgG replacement therapy (IgG-RT) in SHG associated with nephrotic syndrome [3]. More studies are needed to determine whether IgG-RT is beneficial for the treatment of SHG associated with nephrotic syndrome.

Our patient had recurrent relapses within two weeks of stopping steroids and was deemed steroid-dependent. She was recommended immunosuppressive medication but refused to start it due to her history of recurrent infections. Fortunately, her immunoglobulin levels have normalized with the treatment of her MCD, and she has now agreed to start immunosuppressive medications if she relapses again.

## Conclusions

The management of steroid-dependent MCD in adults can be challenging, particularly when complicated by secondary hypogammaglobulinemia, due to the increased risk of infections associated with immunosuppressive therapy. Long-term treatment with steroids requires balancing the maintenance of remission with the minimization of adverse effects. This case highlights the importance of individualized treatment plans for patients at higher risk of infections. Further studies are needed to better understand the long-term management of MCD in adults and the role of IgG-RT in secondary hypogammaglobulinemia.
